# Vernalization Requirement, but Not Post-Vernalization Day Length, Conditions Flowering in Carrot (*Daucus carota* L.)

**DOI:** 10.3390/plants11081075

**Published:** 2022-04-15

**Authors:** Josefina Wohlfeiler, María Soledad Alessandro, Andrés Morales, Pablo Federico Cavagnaro, Claudio Rómulo Galmarini

**Affiliations:** 1 Estación Experimental Agropecuaria La Consulta, Instituto Nacional de Tecnología Agropecuaria (INTA), ex Ruta 40 Km 96, La Consulta 5567, Argentina; josewohlfeiler@gmail.com (J.W.); alessandro.maria@inta.gob.ar (M.S.A.); morales.andres@inta.gob.ar (A.M.); cavagnaro.pablo@inta.gob.ar (P.F.C.); 2 Consejo Nacional de Investigaciones Científicas y Técnicas (CONICET), Buenos Aires 1425, Argentina; 3 Facultad de Ciencias Agrarias, Universidad Nacional de Cuyo, Almirante Brown 500, Luján de Cuyo 5505, Argentina

**Keywords:** flowering, vernalization requirement, photoperiod, annual, biennial, *Daucus carota* L.

## Abstract

Carrots require a certain number of cold hours to become vernalized and proceed to the reproductive stage, and this phenomenon is genotype-dependent. Annual carrots require less cold than biennials to flower; however, quantitative variation within annuals and biennials also exists, defining a gradient for vernalization requirement (VR). The flowering response of carrots to day length, after vernalization has occurred, is controversial. This vegetable has been described both as a long-day and a neutral-day species. The objective of this study was to evaluate flowering time and frequency in response to different cold treatments and photoperiod regimes in various carrot genotypes. To this end, three annual genotypes from India, Brazil, and Pakistan, and a biennial carrot from Japan, were exposed to 7.5 °C during 30, 60, 90, or 120 days, and then transferred to either long day (LD) or short day (SD) conditions. Significant variation (*p* < 0.05) among the carrot genotypes and among cold treatments were found, with increased flowering rates and earlier onset of flowering being associated with longer cold exposures. No significant differences in response to photoperiod were found, suggesting that post-vernalization day length does not influence carrot flowering. These findings will likely impact carrot breeding and production of both root and seed, helping in the selection of adequate genotypes and sowing dates to manage cold exposure and day-length for different production purposes.

## 1. Introduction

The proper time for flowering is crucial for successful reproduction since, in many plant species, the transition of the meristem from vegetative to reproductive development is irreversible [1]. Thus, plants have evolved intricate signaling networks to sense and monitor photoperiod and temperature changes to control flowering time. Prolonged exposure to cold temperatures, which leads to vernalization, is a flower-promoting signal that many plants in temperate regions use to ensure that flowering occurs under more favorable environmental conditions of the spring, while photoperiodism is the physiological response to day length [2,3].

Carrots require vernalization to induce flowering [4,5], and they are classified as early flowering, also called annuals; or late flowering, also called biennials. Wild carrots are usually annual and most cultivated carrots are biennial [4], although there are some annual commercial cultivars adapted to warmer climates [6]. Carrot plants are sensitive to vernalization after a juvenile period has ended, and this stage culminates when the carrot holds 8–12 leaves and storage roots are greater than 4–8 mm in diameter [7,8,9]. Variable time lengths of exposure to cold temperature are required for carrot vernalization, and such a time threshold is genotype-dependent. In general, biennial cultivars require a period of 11–12 weeks at 5 °C to flower [7,10], whereas annuals need shorter periods of cold exposure, from 5 to 30 days [9,11]. In addition, variation within annuals and biennials has been reported, suggesting a gradient of vernalization requirement (VR) in the carrot germplasm [12]. Alessandro and Galmarini [13] examined VR in F_1_, F_2_, and BC_1_ progenies derived from a cross between the annual cultivar ‘Criolla INTA’ and a biennial inbred line from the Argentine carrot breeding program at INTA, and reported that carrot VR was a monogenic trait, with the annual habit being dominant over biennial. This simply inherited trait, termed *Vrn1*, was genetically mapped onto chromosome 2 using an F_2_ family of the same genetic background [14]. However, a subsequent study by the same group using F_2_ populations derived from crosses among diverse genetic backgrounds revealed a model of two genes and three alleles for the control of this trait, with dominance of annuality for both genes [15].

In carrots, vernalization requirement is an important trait for both root and seed production. Bolting is detrimental for commercial root production as the xylem lignifies immediately after the plant has been vernalized, leading to reduced consumer quality (the root becomes fibrous and unpalatable) and loss of commercial value [5,16]. For seed production, VR strongly influences the time/dates of flowering, seed set, and harvest; therefore, intra-cultivar uniformity for VR is highly desirable. Thus, because of the above, the flowering habit of a carrot cultivar determines its sowing date, depending on whether the production purpose is seed or roots.

The effect of day length after vernalization in cultivated carrot is unclear. The first studies stated that vernalized carrot plants were not influenced by the photoperiod, with regards to flowering time [10,17]. Conversely, according to Atherton et al. [18], after the vernalization period long days were needed to allow floral stem elongation and subsequent flowering in a Chantenay type carrot. These contradicting results make it difficult to define carrot as a neutral-day or long-day species with regards to its flowering photoperiod response. Thus, the objective of the present study was to evaluate flowering time and frequency in response to different cold treatments and post-vernalization photoperiod regimes in various annual and biennial carrot genotypes.

## 2. Results

No flowering was observed in control treatment plants grown under constant LD without cold exposure, suggesting that vernalization is required for carrot flowering. Significant variation was found for flowering frequency (%) among the different chilling treatments (*p* < 2.2 × 10^−16^) and among genotypes (*p* = 2.0 × 10^−8^), whereas no significant differences were obtained between the two post-vernalization photoperiod treatments (i.e., LD vs. SD) (*p* = 0.52) (Table 1). Significant variation was also found for flowering time among the cold treatments (*p* = 2.0 × 10^−7^) and genotypes (*p* < 2.0 × 10^−16^), but no significant variation was found between photoperiod regimes (*p* = 0.5), while the interaction between cold treatment and photoperiod was significant (*p* = 0.016), with the rest of the interactions being not significant (Table 1).

**Figure 1:**
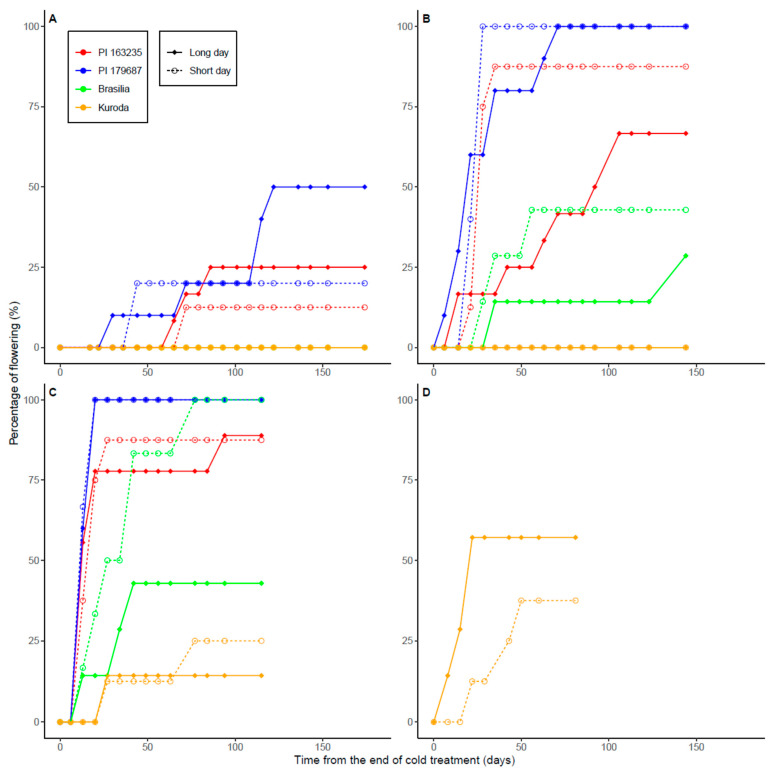
Percentage of flowering in the annual carrot accessions PI179687 and PI163235, the annual cultivar Brasilia, and the biennial cultivar Kuroda after 30 (**A**), 60 (**B**), 90 (**C**), and 120 (**D**) days of cold exposure (mean daily temperature was 7.53 °C). No flowering was observed in control treatment plants grown at ~23 °C and LD (data not shown).

After 30 days of chilling, flowering was observed in the annual accessions PI179687 (flowering ratio was 50% with LD and 20% with SD) and PI163235 (25% with LD and 12.5% with SD), whereas the annual Brasilia and the biennial Kuroda revealed no flowering (Figure 1A). PI179687 started flowering 30 (LD) and 44 (SD) days after the chilling treatment (DACT) and reached its maximum 122 and 44 DACT, respectively; PI163235 initiated flowering at days 65 (LD) and 72 (SD) and reached a plateau at days 86 and 72 under LD and SD, respectively.

When exposed to 60 days of cold, PI179687 reached 100% of flowering in both photoperiod treatments; PI163235 reached 87.5% flowering under SD and 66.7% under LD and Brasilia reached 42.9% flowering under SD and 28.6% under LD (Figure 1B). These three annual genotypes reached their maximum flowering ratios sooner under SD than LD; for PI163235 and Brasilia, greater overall flowering ratios were attained under SD. Thus, PI179687 began flowering 6 and 21 DACT for LD and SD, and reached their maximums 71 and 28 DACT, respectively. For PI163235, flowering initiation was 14 and 21 DACT for LD and SD, reaching their respective plateaus of 106 and 35 DACT; for Brasilia, flowering began 35 and 28 DACT for LD and SD, with their respective maximums being 144 and 56 DACT. In contrast to the three annual genotypes, the biennial cultivar Kuroda did not flower at all under both LD and SD treatments.

After 90 days of cold treatment, all the carrot genotypes evaluated had some percentage of flowering plants and exhibited clear differences in their time-course progression curves and maximum flowering ratios (Figure 1C). In decreasing order, PI179687 reached 100% flowering under both LD and SD conditions; PI163235 reached comparable maximums under LD (88.9%) and SD (87.5%); Brasilia attained 100% flowering under SD and 42.9% under LD, and Kuroda presented the lowest—yet comparable—maximums of 25% and 14.3% under SD and LD, respectively. Initiation of flowering for the annual genotypes PI179687, PI163235, and Brasilia took place between the first and the second week after the end of the cold treatment, reaching maximum flowering ratios 20 DACT for PI179687 in both photoperiod treatments, 27 DACT for PI163235 under SD, 94 DACT for PI163235 under LD, 42 DACT for Brasilia under LD, and 77 DACT for Brasilia under SD. Flowering initiation in the biennial Kuroda occurred later than in the annual genotypes, namely between the 3rd and 4th week after the cold treatment for both photoperiod conditions, after which little or no increment in flowering ratio was observed.

Given its apparently greater vernalization requirement, as compared to the annual genotypes, Kuroda was further tested using 120 days of cold exposure (Figure 1D). Under these conditions, flowering began the first week under LD conditions and between the 2nd and 3rd week under SD, reaching maximum flowering ratios of 57.1% and 37.5% for LD and SD, respectively.

The length of the cold treatment and flowering frequencies were significantly (*p* < 0.05) and strongly correlated in all of the carrot germplasm evaluated, as indicated by the high normalized point biserial correlation coefficient values obtained between these two variables in PI179687 (r = 0.91–0.92), PI163235 (r = 0.75), Brasilia (r = 0.69–0.95), and Kuroda (r = 0.86–0.67) (Table S1, supplementary materials).

Flowering time was significantly affected by the carrot genotype (*p* < 2 × 10^−16^), the cold treatment (*p* = 2.0 × 10^−7^), and the interaction between the cold treatment and photoperiod (*p* = 0.016), but not by the post-vernalization photoperiodic conditions (*p* = 0.5) (Table 1). For all the carrot genotypes evaluated, the duration from the end of the cold treatment to the elongation of the first floral stem internode, considered as the beginning of flowering, decreased as the length of the cold treatment increased, regardless of the post-vernalization photoperiodic conditions (Figure 1). Thus, for PI179687, after 30 days of cold exposure, the first floral stem elongated 30–44 DACT, after 60 days of cold exposure, flowering initiated 6–21 DACT, and after 90 days of cold, flowering began 13 DACT. Similarly, under 30, 60, and 90 days of cold treatment, PI163235 initiated flowering 65–72, 12–21, and 13 DACT, respectively, whereas for Brasilia, which only generated flowering plants after 60 and 90 days of cold exposure, flowering began 28–35 and 13 DACT, respectively. In the case of the biennial Kuroda, which produced flowering plants only after 90 and 120 days of cold, flowering initiation was 27 and 8–22 days after these cold treatments, respectively. Altogether, the inverse relationship between the length of the cold treatment and flowering time (scored as the number of days after the cold treatment required for initiation of flowering) was further evidenced by the significant (*p* < 0.05) and strong negative Pearson correlation values found between these two variables for PI179687 (r = −0.70 to −0.90), PI163235 (r = −0.62 to −0.85), Brasilia (r = −0.63 to −0.18), and Kuroda (r = −0.62 to −0.25) (Table S2, Supplementary Materials).

## 3. Discussion

We found clear differences in vernalization requirements among the four carrot genotypes evaluated, varying from very low VR in the annual accession PI179687 to high VR in the biennial cultivar Kuroda (Figure 1). The carrot accessions PI179687 and PI163235, originally from India and Pakistan (in the Southern border of Central Asia, the center of origin of carrot), had the lowest vernalization requirements and shortest flowering times after cold exposure. PI179687 yielded the greatest flowering frequency (with up to 50% flowering after 30 days of cold, and 100% flowering after 60 and 90 days of cold), followed by PI163235 (with up to 25%, 87.5%, and 88.9% of flowering after 30, 60, and 90 days of chilling, respectively), and Brasilia (with up to 45% and 100% flowering after 60 and 90 days of cold exposure, respectively). Lastly, the Japanese biennial cultivar Kuroda revealed no flowering plants when exposed to 60 or less days of chilling, whereas with 90 and 120 days of cold, up to 25% and 57.1% of the plants flowered, respectively (Figure 1). The model that predicts flowering frequency in response to the length of the cold treatment states that flowering frequency increases with the length of the cold treatment in a logistic regression manner, and the responses vary among the genotypes in accordance with their VR (Figure S1, Supplementary Materials). The fact that only vernalized plants were able to flower (i.e., none of the Control plants without cold treatment flowered) strongly suggests that VR in carrot is obligate, as defined by Michaels and Amasino [1] and coincides with previous results in this species [12]. These results are also supported by this model (Figure S1), predicting that under 0 days of cold, no flowering is expected.

Concerning the geographical origin of the carrot germplasms used, our data suggest that carrots from tropical or subtropical regions tend to have lower VR and, consequently, shorter flowering time and greater flowering frequencies than carrots from temperate regions. These results are consistent with Central Asia as the proposed region for carrot domestication [19] and the hypothesis that during domestication in temperate regions carrots may have lost their tendency for premature flowering [5].

Brasilia and Kuroda were previously evaluated by Wohlfeiler et al. [12]. Comparing the vernalizing temperature used in the present study (~7.5 °C) with that of our previous work (5.6 °C), both treated with LD after the cold treatment, herein we observed that Brasilia revealed no flowering after 30 days of cold, 29% after 60 days of cold, and 43% after 90 days of cold, whereas in our previous work (at 5.6 °C) it showed 0% after 30 days of cold, 64% (60 days), and 93% (90 days) flowering. Similarly, Kuroda did not flower (0% flowering) under 30 and 60 days of cold treatment at these two temperatures, whereas it revealed 14% and 43% of flowering after 90 days of cold treatment at 7.5 °C and 5.6 °C, respectively. Altogether, these data reinforce our previous hypothesis that increased flowering rates are associated with more intense cold exposures [12].

The results of the present study confirm the gradient of vernalization requirement reported recently among different carrot germplasm [12]. In this previous study we proposed genotypes for the two loci conditioning VR in these germplasms and hypothesized a certain number of hours of cold exposure that each vernalization allele requires for the plant to become vernalized [15]. In agreement with the genetic model proposed, in the present study, Kuroda was the latest to flower among all the cultivars evaluated, yielding only 14.3% and 25% of flowering after 90 days of cold treatment, and 57.1 and 37.5 after 120 days of cold with LD and SD, respectively. On the other hand, Brasilia presented an annual flowering habit, although it is not as extreme as other early flowering genotypes. This cultivar revealed 28.6% and 42.9% of flowering after 60 days of cold, and 42.9% and 100% after 90 days of cold, with LD and SD, respectively. From results of the present study, we now hypothesize that PI179687 and PI163235, which have not been evaluated previously, harbor different early flowering alleles conditioning the lowest VR, the shortest flowering cycle, and the greatest flowering frequencies reported to date among the carrot germplasm. 

Previous studies have reported that vernalization accelerated the initiation of flowering in carrot [7,11,17,20,21] and other plant species [1,2]. In the present study, longer cold treatments promoted not only earlier flowering initiation, but also greater maximum flowering rates attained (Figure 1). This last aspect is of crucial importance for carrot seed production and suggests that regions with long winters will result in higher seed production yields. Alternatively, if carrot roots are vernalized in cold storage, our data suggest that extending the cold treatment for up to 3–4 months would result in high flowering frequencies and, thereby, high seed production for most carrot genotypes. The model that predicts flowering time in response to the duration of the cold and photoperiod treatments states that flowering time decreases with the length of the cold treatment in a log-linear manner, and the responses vary among the genotypes in accordance with their VR (Figure S2, supplementary materials).

Post-vernalization photoperiod conditions had no significant effect on neither flowering frequency nor flowering time, suggesting that carrot is a neutral species with regards to its flowering response to day length after it has been vernalized. These results are in agreement with previous classifications of carrot as a neutral-day species by Sakr and Thompson [17] and Hiller and Kelly [10] and contradict results of Atherton et al. [18] indicating carrot as a long-day species. It also contradicts another root crop species as *Beta vulgaris* since it is considered as a long-day species [22]. In these previous studies the carrot genotypes used were not specified, and therefore, direct comparisons across the studies are not possible. A possible explanation for these discrepancies could be that genotypes with different post-vernalization photoperiod responses exist in the carrot germplasm, including long-day and neutral-day materials, and perhaps such genotypic variants were used in these studies. Different photoperiod responses could have arisen as consequence of different domestication histories and selection pressures in distinct or contrasting environments. From an evolutionary point of view, having no photoperiod requirements for flowering represents an adaptive advantage, as the plant can leave offspring all year round, even when days are getting shorter in autumn. This lack of photoperiod requirement may also represent an advantage for carrot breeders that often induce vernalization by storing roots in refrigerated chambers, since once vernalized, the roots can be immediately transplanted in the field regardless of the current photoperiod.

## 4. Materials and Methods

Four carrot genotypes from diverse geographical origins were used in this study and included three annual carrots (two germplasm accessions and a commercial cultivar) and one biennial cultivar. Table 2 presents information on the flowering habit, geographic origin, genetic structure, and seed source of the four genotypes.

Seeds of the annual carrot genotypes PI179687 (from India), PI163235 (from Pakistan), and Brasilia (from Brazil), and the biennial cultivar Kuroda (from Japan) (Table 2) were sown in pots (10 cm diameter × 18 cm high) containing fertile soil (14% organic matter, pH 6.8, and a carbon-to-nitrogen ratio of 15) in a growth chamber (a room with controlled temperature using air conditioning and heating). The carrots were sown on 6 June 2019, and grown under the following conditions: mean day temperature: 21.7 °C, long day photoperiod, 16 h of light (light intensity: 166.6 µmol m^−^^2^ s^−^^1^), and 8 h of darkness (Table 3). When the plants had three unfolded true leaves [23] they were thinned to one individual per pot. On 10 October 2019, when the plants held 8–12 leaves juvenility was assumed to have ended [7,8,9] the different chilling treatments were applied to each of the carrot genotypes using a completely randomized design. For the vernalization treatments, a mean day temperature of 7.53 °C was applied during 30, 60, 90, and 120 (only for Kuroda) days under short day conditions (8 h of light and 16 h of darkness) (Table 3). Seven individuals of each genotype remained in the growing chamber at 21.7 °C under long-day regime as controls. When each chilling treatment finished, the pots were transferred to post-vernalization chambers; half of them with long day (LD) photoperiod and the other half with short day (SD) photoperiod (Table 3).

During the whole experiment, the plants were watered twice a week, and the pots were arranged completely at random within each chamber to eliminate any location effect. An individual was considered to have flowered when the first floral stem internode elongated, as proposed by Alessandro and Galmarini [13]. Flowering was recorded at 1 week intervals for 20 weeks after the end of the chilling treatments. Percentage of flowered plants (henceforth referred to as ‘flowering frequency’) for each cultivar, and number of days from the end of chilling treatment (henceforth ‘flowering time’), were calculated. To describe the relationship between the length of the cold treatment and both flowering frequency and flowering time, we performed regression analysis between the first variable and the flowering response variables. The relationship between length of the cold treatment and flowering frequency was not linear, and followed a logistic function, and therefore logistic analysis of covariance, namely a binomial Generalized Linear Model (GLM), was applied. In the case of flowering time, the relationship with length of the cold treatment was log-linear. Thus, given that flowering time follows a log-normal distribution, a linear regression analysis between ‘length of the cold treatment’ and the logarithm of flowering time was performed. On both analyses, the length of the cold treatment was considered as a covariate, since modelling it as a fixed factor did not translate into a statistically different fit. All statistical analyses were performed using package ‘stats’ in R version 4.1.2 [24].

## 5. Conclusions

Understanding VR is a major goal for carrot breeding, as early bolting can cause a significant decrease in marketable root yield due to lignification of the root after the plant has been vernalized. In particular, precise knowledge on the environmental and genetic factors influencing this trait is crucial for the selection of adequate plant materials (i.e., carrot cultivars) and sowing dates across different environments and end-purposes (i.e., production of roots or seeds). Results from the present study using additional carrot genotypes in independently-conducted experiments confirm our previous findings [12,15], indicating that: (1) a quantitative gradient of vernalization requirement exists in the carrot germplasm, presumably conditioned by multiple VR genes; (2) the length and intensity of the cold treatment accelerates initiation of flowering and increases flowering frequency; (3) early flowering (annual habit) is dominant over late flowering (biennial habit). In addition, this study characterized VR, flowering time, and flowering frequency in two new early-flowering accessions, namely PI179687 and PI163235, these being the carrot genotypes with the lowest VR reported to date.

The present study evaluated, for the first time, the effect of post-vernalization photoperiodic conditions on both ‘flowering time’ and ‘flowering frequency’ in four genetically and geographically diverse carrot genotypes (three annual and one biennial), representing the most comprehensive analysis reported to date in terms of number of genotypes, conditions, and variables analyzed. Our results demonstrate that vernalization, but not post-vernalization photoperiod, influences flowering frequency and flowering time in these germplasms. These results suggest that carrot is a ‘neutral-day’ species with regards to its photoperiod response for flowering. However, considering the previous studies reported long days as a post-vernalization requirement for flowering in carrot genotypes, different from the ones used in this study, we cannot rule out intraspecific variation for this trait and the existence, perhaps, of neutral-day and long-day germplasms. In the future, comparison of genotypes previously reported to be long-day or neutral-day, under the same experimental conditions, would provide conclusive evidence on the flowering response of carrot to post-vernalization photoperiod.

## Figures and Tables

**Table 1:** Effect of cold treatment (CT), post-vernalization photoperiod (Ph), genotype (G), and their interactions on flowering time and frequency in carrot.

Dependent Variable	Factor	d.f.	Deviance/F ^a^	*p*
Flowering frequency	**Cold treatment**	**1**	**105.9**	**<2.2 × 10^−16^**
	Photoperiod	1	0.41	0.52
	**Genotype**	**3**	**38.7**	**2.0 × 10^−8^**
	CT × Ph	3	0.31	0.58
	CT × G	3	5.9	0.12
	Ph × G	3	5.0	0.17
	CT × Ph × G	3	3.6	0.31
log(Flowering time)	**Cold treatment**	**1**	**33.2**	**2.0 × 10^−7^**
	Photoperiod	1	0.55	0.50
	**Genotype**	**3**	**7.7**	**<2.0 × 10^−16^**
	**CT × Ph**	**3**	**6.1**	**0.016**
	CT × G	3	0.23	0.90
	Ph × G	3	0.73	0.54
	CT × Ph × G	3	0.32	0.85

Significant values are indicated in bold (*p* < 0.05). ^a^ Numbers are deviance from binomial Generalized Linear Model (GLM) for flowering frequency, and F values from ANOVA for log (Flowering time); d.f. degrees of freedom.

**Table 2:** Characteristics of the four carrot genotypes used in this study.

	Accession/Cultivar Name			
	PI 179687 ^a^	PI 163235	Brasilia	Kuroda
Genetic structure	landrace	landrace	OP ^b^	hybrid
Geographical origin	India	Pakistan	Brazil	Japan
Flowering habit	annual	annual	annual	biennial
Seed source	USDA ^c^	USDA	USDA	Private company (Sakata)

^a^ Accession ID of the plant introduction at the Germplasm Resources Information Network (GRIN)—United States Department of Agriculture (USDA) database. ^b^ OP. Open pollinated. ^c^ USDA: United States Department of Agriculture.

**Table 3:** Temperature, photoperiod, and light intensity conditions in each chamber used for carrot growing.

	Growing Chamber	Vernalization Chamber	LD Post-Vernalization Chamber	SD Post-Vernalization Chamber
Minimum temperature ^a^ (°C)	15.0	5.0	19.5	13.0
Maximum temperature (°C)	25.5	19.0	33.5	26.0
Mean temperature (°C)	21.71	7.53	25.0	22.92
CV of temperature (%)	7.24	31.1	6.63	32.9
Photoperiod	LD ^b^	SD ^b^	LD	SD
Average light intensity (µmol m^−2^ s^−1^)	166.6	140.6	166.6	140.3

^a^ Temperatures in the chambers were measured every hour with iButton Thermochron DS1921G data logger. ^b^ LD: 16 h of light and 8 h of darkness; SD: 8 h of light and 16 h of darkness.

## Data Availability

All data included in the main text and supplementary materials.

## Supplementary Materials

The following supporting information can be downloaded at: https://www.mdpi.com/article/10.3390/plants11081075/s1, Table S1: Normalized point biserial correlation coefficient values between the length of the cold treatment and flowering frequency for each carrot genotype and post-vernalization photoperiod treatment; Table S2: Pearson correlation coefficient values between the length of the cold treatment and log-transformed flowering time for each carrot genotype and post-vernalization photoperiod treatment; Figure S1: Predicted values and 95% confidence interval (shaded area) for flowering frequency in response to the duration of the cold treatment. Observed data are shown in isolated circles (SD) and diamond-shape figures (LD); Figure S2: Predicted values and 95% confidence interval (shaded area) for flowering time in response to the duration of the cold treatment and photoperiod treatment. Observed data are shown in isolated circles (SD) and diamond-shape figures (LD).
