# Supplementary material for: Vernalization Requirement, but Not Post-Vernalization Day Length, Conditions Flowering in Carrot (Daucus carota L.)
Source: Plants (Basel). 2022 Apr 15;11(8):1075. doi: 10.3390/plants11081075 (PMC9029871; doi:10.3390/plants11081075)
Supplement: Supplementary file 1 [file plants-11-01075-s001.zip › Supplemental-Tables-S1-and-S2.pdf]

Vernalization requirement, but not post-vernalization day length, conditions flowering in carrot (*Daucus carota* L.)

Josefina Wohlfeiler, María S. Alessandro, Andrés Morales, Pablo F. Cavagnaro and Claudio R. Galmarini.

Table S1. Normalized point biserial correlation coefficient values between the length of the cold treatment and flowering frequency for each carrot genotype and post-vernalization photoperiod treatment.

Days of cold treatment	PI179687		PI163235		Brasilia		Kuroda	
	LD	SD	LD	SD	LD	SD	LD	SD
0	0	-	0	-	0	-	0	-
30	50	20	25	12.5	0	0	0	0
60	100	100	66.7	87.5	28.6	42.9	0	0
90	100	100	88.9	87.5	42.9	100	14.3	25
120	-	-	-	-	-	-	57.1	37.5
Corr. coef. (r)[a]	0.91	0.92	0.75	0.75	0.69	0.95	0.86	0.67

Values correspond to the percentage of flowering. Dashes (-) indicate lack of flowering plants or untested treatment. LD. Long-day (16 hours of light and 8 hours of darkness); SD. Short-day (8 hours of light and 16 hours of darkness). [a] Normalized point biserial correlation coefficient.

Table S2. Pearson correlation coefficient values between the length of the cold treatment and log-transformed flowering time for each carrot genotype and post-vernalization photoperiod treatment.

Days of cold treatment	PI179687		PI163235		Brasilia		Kuroda	
	LD	SD	LD	SD	LD	SD	LD	SD
30	90.8	44	74.3	72	-	-	-	-
60	30.1	25.2	63.5	28	89.5	39.7	-	-
90	15.8	15.3	24.9	18	29.7	36.8	27	52
120	-	-	-	-	-	-	16.8	38.3
Corr. coef. (r)[a]	-0.70	-0.90	-0.62	-0.85	-0.63	-0.18	-0.62	-0.25

Values correspond to the mean number of days after the cold treatment (DACT) until initiation of flowering. Dashes (-) indicate lack of flowering plants or untested treatment. LD. Long-day (16 hours of light and 8 hours of darkness); SD. Short-day (8 hours of light and 16 hours of darkness). [a] Pearson correlation coefficient.
