# Supplementary figures and images for: Vernalization Requirement, but Not Post-Vernalization Day Length, Conditions Flowering in Carrot (Daucus carota L.)
Source: Plants (Basel). 2022 Apr 15;11(8):1075. doi: 10.3390/plants11081075 (PMC9029871; doi:10.3390/plants11081075)

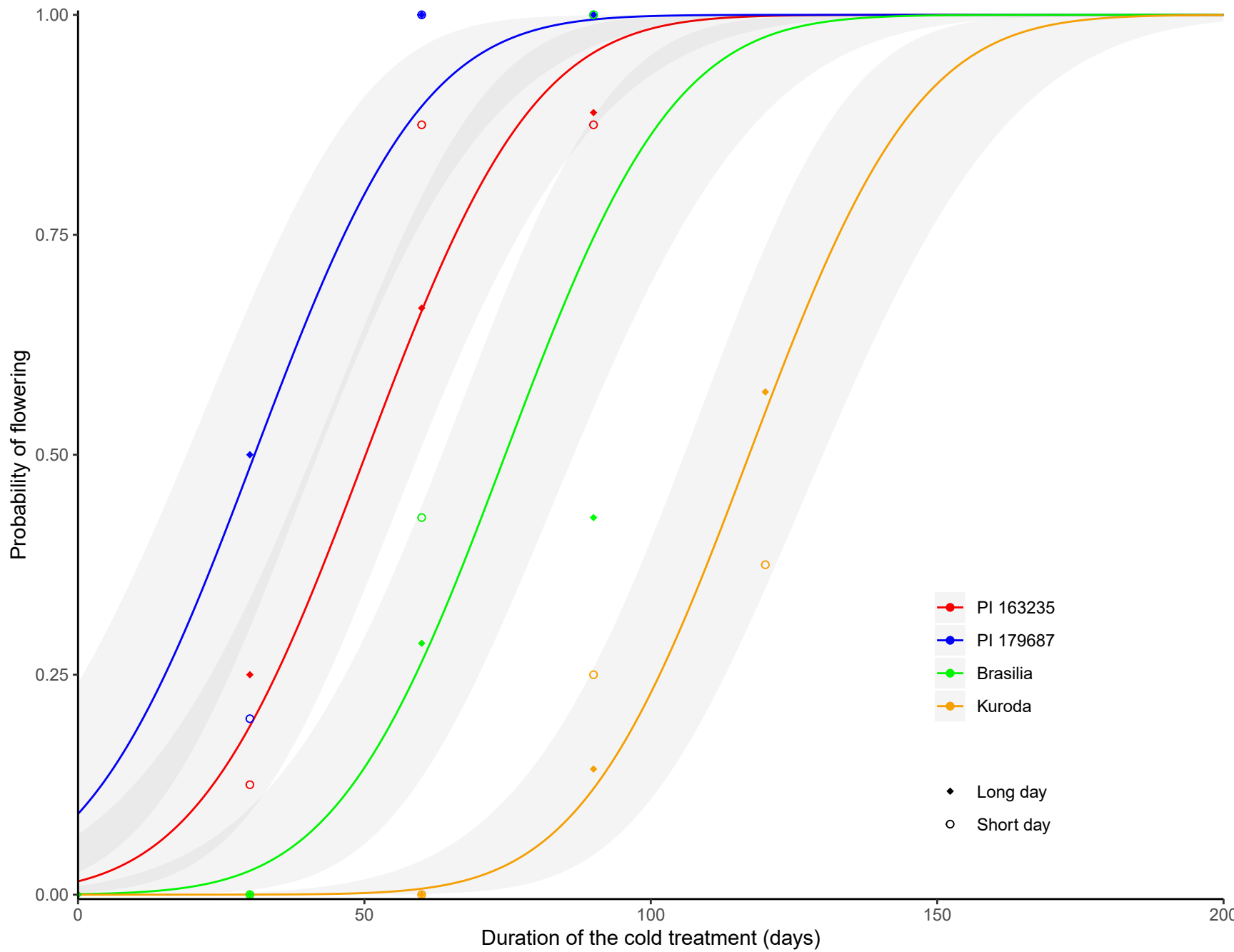

Supplement: Supplementary file 1 [file plants-11-01075-s001.zip › Figure-S1.pdf]

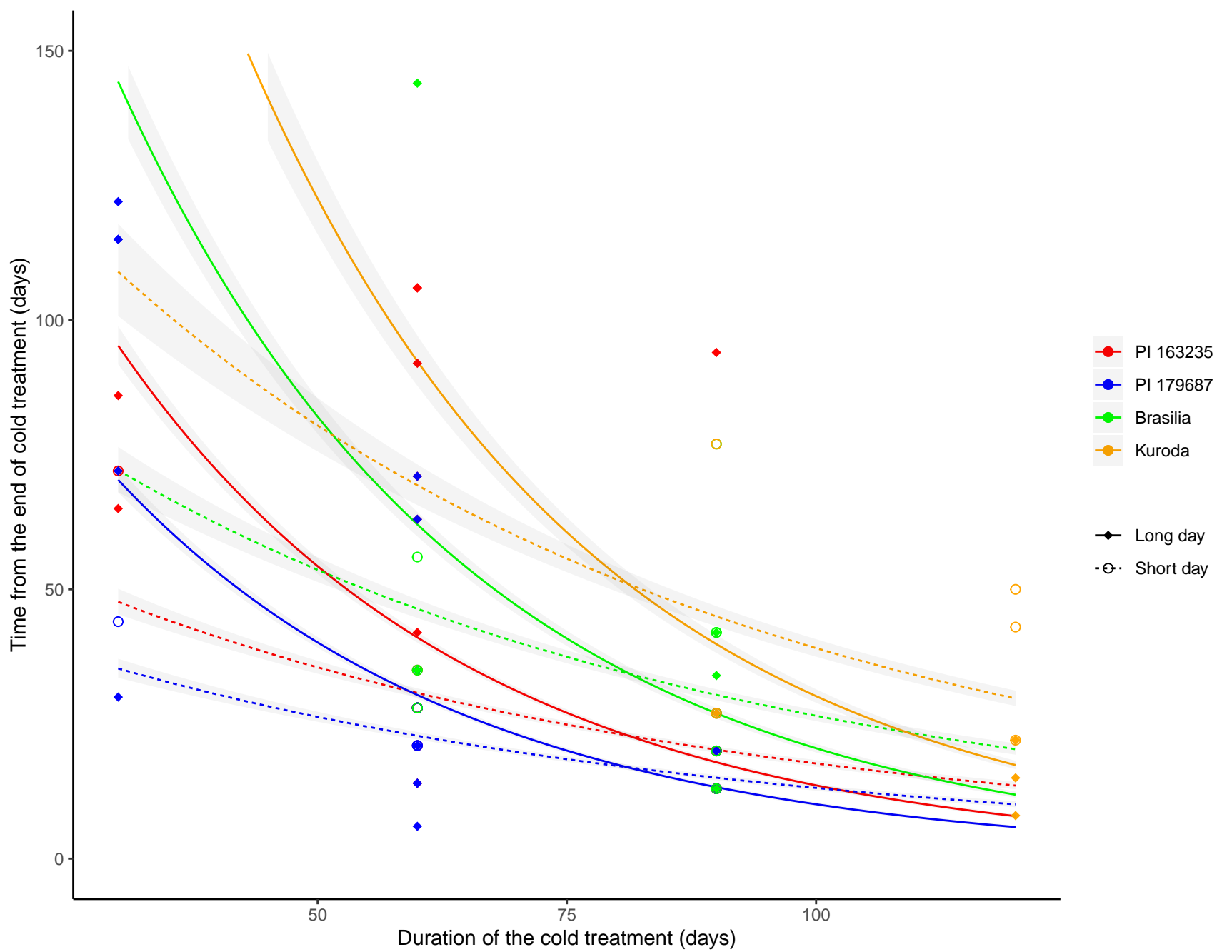

Supplement: Supplementary file 1 [file plants-11-01075-s001.zip › Figure-S2.pdf]
